# 

**Published:** 1825-01-01

**Authors:** 


### THE MEDICO-CHIRURGICAL REVIEW.

*NEW SERIES.*

### VOLUME TWO.

[BEING VOL. VI. OF ANALYTICAL SERIES.]

CONDUCTED BY

ASSOCIATED PHYSICIANS AND SURGEONS;

AND SUPERINTENDED BY

JAMES JOHNSON, M.D.

MEMBER OF THE ROYAL COLLEGE OF PHYSICIANS OF LONDON, AND PHYSICIAN EXTRAORDINARY TO HIS ROYAL HIGHNESS THE DUKE OF CLARENCE.

Nec tibi quid liceat sed quid fecisse decebit

Occurrat mentemque domat respectus honesti.—CLAUD.

LONDON:

*Printed by G. Hayden, Little College Street, Westminster.*

Published by Burgess & Hill, Windmill Street, Haymarket; Baldwin, Cradock, and Joy, Pater-Noster Row; Kingsbury, Parbury, & Allen, Leadenhall Street; Highley, and T. & G. Underwood, Fleet Street; Callow & Wilson, Princes Street, Soho; Cox & Son, Borough; Jackson, Borough; Anderson, West Smithfield; John Cox, Berners Street, Oxford Street; Adam Black, Edinburgh; Richard Griffin & Co. Glasgow; Hodges and M'Arthur, Dublin; Messrs. Treuttel & Wurtz, Paris and Strasburg.

1825.

### BIBLIOGRAPHICAL RECORD;

OR,

### *Works received for Review since last Quarter.*

1. Essays on Various Subjects of Medical Science. By David Hossack, M.D. F.R.S. L. & E. &c. &c. 2 vols. 8vo. pp. 472. New York, 1824.

☞ *See this Number of our Review.*

---

2. Practical Observations on the Lateral Operation of Lithotomy; and on Various improved and new modes of performing this operation; together with Remarks on the Recto Vesical Operation. By Robert Muter, M.D. Member of the Royal College of Surgeons in London, Author of "Observations on Various Novel Modes of operating on Cataract," &c. 8vo. pp. 107, New York, 1824.

---

3. Practical Observations on Hydrophobia, with a Review of the Remedies employed, and Suggestions for a different Treatment of that Disease. By John Booth, M.D. &c. &c. &c. 8vo, stitched, pp. 16, London, 1824.

---

4. Inaugural Address delivered before the Medical Society of the County of New York, on the 12th day of July, 1824. By David Hossack, M.D. LL.D. President of the Society.

☞ *Dr. Hossack reviews, with equal ability and earnestness, the great objects of the Society.—the encouragemeet and cultivation of good Medical Ethics—the discouragement of every thing savouring of Charlatannerie—the prosecution of Medical and Chirurgical Science—and last, not least, a pious regard to the Memory of their departed brethren, as manifested in Biographical Records of their virtues and good deeds. This is all done in a tone of the very best feeling, and it must have made an adequate impression on the minds of the assembled auditors.*

---

5. A Practical Treatise on Hæmorrhoids or Piles, Strictures, and other important Diseases of the Rectum and Anus; being, with some additions, a Treatise to which the Jacksonian Prize was adjudged by the Royal College of Surgeons. By George Calvert, Member of the Royal College of Surgeons of London, &c. 8vo. pp. 359. London, 1824. ☞ *In our next.*

---

6. Observations illustrative of the Nature and Treatment of the prevailing Disorders of the Stomach and Liver. By Thomas John Graham, M.D. M.R.C.S. 8vo. pp. 224. London, 1824.

---

7. Manuale Medicum, or a Medical Pocket-book, for the use of Students. Adapted to the last Edition of the Pharmacopœia Londinensis. By H. L. Sanders, Surgeon. Small 8vo. pp. 200, Callow and Wilson, Sept. 1824.

☞ *In this little Work, we recognize the plan and much of the substance of Dr. George Pearson's Lectures. It comprises a great mass of elementary and standard matter in a small space, and lucid order. It is divided into two parts; the first containing the various articles of the Materia Alimentaria and Medica, arranged into classes, followed by their doses, and mode of exhibition. The second part relates to Diseases, and is arranged into classes, orders, &c containing briefly the character, symptoms, diagnosis, prognosis, causes, and treatment of the more important Disorders of this Country, rendered into Latin, and adapted to the Cullenian arrangement. It will prove a useful manual to the student, and to those who are to undergo examinations before the constituted Authorities.*

---

8. Dissertatio Inauguralis quædam de Topographia Medica Complectens. Thomas A. Wise, M.D. Auctor.

☞ *A very creditable Thesis.*

---

9. Practical Remarks. Part 1.—On Acute and Chronic Ophthalmia, Ulcers of the Eye, &c. &c. Part 2.—On Remittent Fever, viz. Simple and Complicated. By Thomas O'Halloran, M.D. Author of "Treatises on the Barcelona and Andalusian Yellow Fever Epidemics. 8vo. pp. 148. London, 1824. ☞ *In our next.*

---

10. Vindication of the introduction in Edinburgh of Vapour and Medical Baths; together with a Letter to the Managers of the Royal Public Dispensary, on what passed at a Meeting for the Institution of a New Dispensary for Diseases of the Skin, and on the Saving that may be made to the Public in their generous and charitable contributions. By William Scott, Esq. 8vo. sewed. Ed. 1824.

☞ *We are sorry to see these appeals to the Public, on local and private clashings of interest. But, alas! Edinburgh has long been famous for these "domestic rows." Were we only to listen to one side of the question, we should suppose Mr. Scott has been hardly treated by his young friend, Dr. Duffin. But, "audi alteram partem," is a maxim from which we hope we shall never swerve.*

---

11. Revue Medicale Francaise et Etrangere, et Journal de Clinique de l'Hotel Dieu, et de la Charité de Paris. Aug. Sept. Oct. and Nov. 1824.

☞ *We congratulate the Editors of this Journal on the increasing fame of their Work. The Revue Medicale is not surpassed by any periodical Journal in Europe or America.*

---

12. Observations on the History, Use, and Construction of Obturateurs, or what have hitherto been called in this country *Artificial Palates;* with cases illustrative of recent improvements. By James Snell, Surgeon-dentist; member of the Royal College of Surgeons in London. 8vo. pp. 52, September, 1824.

---

13. Concise Narrative of an Ophthalmia, which prevailed in a detatchment of His Majesty's 44th Regiment, on their Voyage to Calcutta, in the Summer of 1822, together with an account of other Tropical Diseases, and their Treatment on board the H. C. Ship, Warren Hastings. By Richard Jones, F. R. C. S. &c. 8vo. stitched, pp. 57. Warwick, 1824.

---

14. Observations on the History and Treatment of the Ophthalmia accompanying the secondary forms of Lues Venerea. Illustrated with Cases. By Thomas Hewson. A.B. member of the Royal College of Surgeons in Ireland; Professor of Materia Medica and Pharmacy to the College; and Surgeon to the Meath Hospital, and County of Dublin Infirmary, &c. &c. &c. 8vo. pp. 117. London, 1824. ☞ *In our next.*

---

15 A Nosological Practice of Physic, embracing Physiology. By George Pearson Dawson, M.D. 8vo. pp. 380. London, 1824.

---

16. System of Anatomical Plates with descriptive Letter-press, by John Lizars, F. R. S. E. Fellow of the Royal College of Surgeons, and Lecturer on Anatomy and Physiology, Edinburgh. Part VI.—Muscles and Joints of the Lower Extremity.

☞ *The sale of this Work has already become prodigious—a proof that it is generally esteemed by the professional Public. We do not say that it is without faults—we mean in the Plates—but, taken altogether, it is one of the best and the cheapest Publications of the kind produced in this country.*

---

17. An Enquiry into the Mode in which Mercurial Ptyalism operates in the Cure of Acute Disease; and whether there exist other Remedies of similar action that may be advantageously substituted for it. By James Arnott, Author of a Work on Diseases of the Urinary Organs. 8vo. sewed, p. 16. Callow and Wilson, October, 1824. ☞ *See Periscope.*

---

18. Lectures on Digestion and Diet. By Charles Turner Thackrah, member of the Royal College of Surgeons of London—of the "Societé de Medecine-pratique," de Paris, &c. Royal Octavo, pp. 156. Longman & Co. October, 1824.

19. A Treatise on the Use of the Natural and Factitious Waters of Carlsbad, Ems, Marienbad, &c. By Dr. F Kreysig, of Dresden, Physician to His Majesty the King of Saxony, &c. Translated from the German by W. F. Bekenn. Part. II. London, 1824. Octavo, pp. 155.

---

20. The Anatomy of the Brain, adapted for the Use of Students, comprising Directions with regard to the Method to be pursued in its Dissection, conformable to the Mode practised by most Anatomists. Epigraph "Lege Memoriæque Mandato." Duodecimo, pp. 162. London, Callow & Wilson, October, 1824.

☞ *This is a concise and convenient* Vade Mecum *for Cerebral Dissections. It contains the* Mode *of dissecting the Brain, as well as an enumeration of its various parts. A short Appendix conveys a slight sketch of Gall and Spurzheim's Method of unravelling this intricate portion of our organization.*

---

21. An Essay on Curvatures and Diseases of the Spine, including all the Forms of Spinal Distortion; to which the Fothergillian Gold Medal was awarded by the Medical Society of London; and presented at a Special General Meeting, on the 3d of May, 1824; with some additions. By R. W. Bampfield, Esq. one of the Surgeons to the Royal Metropolitan Infirmary for Diseases of Children—Fellow of the Medical Society of London—Author of a Practical Treatise on Tropical and Scorbutic Dysentery, &c. 8vo, pp. 387. London, October, 1824.

☞ *See the present Number of this Review.*

---

22. Outlines of a System of Medico-chirurgical Education, containing Illustrations of the application of Anatomy, Physiology, and other Sciences, to the principal practical points in Medicine and Surgery. By Thomas Turner, member of the Royal College of Surgeons, of London; Lecturer on Anatomy, &c. 8vo, pp. 369, with coloured Plates. Underwoods, Oct. 1824.

☞ *This is not a Work on Medical Education, in the common acceptation of the term. It is, in fact, an Epitome or Outline of Anatomy and Physiology, with slight glances at Pathology, Surgery, and Therapeutics. In this sense, it may be useful to students, as an Index to the subjects which they ought to study. It has nothing to do with any Plan of Studies.*

---

23. Hints towards the adoption of an Improved Principle of remunerating the Surgeon-Apothecary, or General Practitioner in Medicine, addressed to the members of the Profession, and the Public at large. By T. M. Greenhow, member of the Royal College of Surgeons, &c. 8vo, pp. 26. Newcastle, November, 1824.

---

24. Clinical Report on Dropsies; with Observations explanatory of their Pathology and Therapeutics; with an Appendix on the Theory and Treatment of Organic Disease in general. By Robert Venables, Bachelor in Medicine, and Licentiate in Physic of the University of Oxford, Physician to the Henley Dispensary, &c. 8vo, pp. 238. Underwoods, Nov. 1824.

---

25. A Treatise on the most celebrated Mineral Waters of Ireland, containing an Account of the Waters of Ballyspillan, Castleconnel, Ballynahinch,

Mallow, Lucan, Swadlinbar, Goldenbridge, Kelmainham, &c. and of the Spa lately discovered at Brownstown, near Kilkenny; with plain Directions during the Use of the Mineral Waters, &c. By Michael Ryan, M.D. Member of the Royal College of Surgeons of London, &c. Octavo, sewed, pp. 45. Kilkenny, November, 1824.

☞ *This little Treatise will prove a useful index to the Medicinal Springs of the Sister Isle.*

---

26. A Map of the Ear; the Subjects taken from Anatomical Preparations in the Possession of John Harrison Curtis, Esq. designed chiefly for the Use of his Pupils.

☞ *In this Plate, which is beautifully coloured, there are nine figures, eight of which delineate the parts separately—and the ninth exhibits a general view of the anatomy of the head in the vicinity of the ear. This Plate is certainly well calculated to facilitate the student's early acquisition in aural anatomy, and to him we can recommend it.*

---

27. Opinions on the Causes and Effects of the Disease denominated Tic Douloureux; deduced from Practical Observations of its supposed Origin in Lateral Pressure, Distortion, or Undue Contact in the Teeth; but more particularly those nearest the Maxillary Sinus, and thence conveying its distressing Sensations to the more distant Extremities of the System. The whole illustrated by Three Lithographic Engravings, with Cases, &c. By Charles Bew, Surgeon-Dentist to His Majesty, &c. Octavo, pp. 94. London, Underwood and Co. December, 1824.

---

28. Philadelphia Journal of the Medical and Physical Sciences, &c. Number 16, for August, 1824. Conducted by Dr. N. Chapman, &c.

*In Exchange.*

☞ *In this Number the erudite and very able Editor has commenced an interesting Memoir on Epidemics.*

---

29. Researches, Physiological and Pathological; instituted principally with a View to the Improvement of Medical and Surgical Practice. By James Blundell, M.D. Lecturer on Physiology and Midwifery at the united Hospitals of St. Thomas and Guy. Octavo, pp. 145, Three Plates. Cox and Son, December, 1824.

☞ *In our next.*

---

*** Note.—Authors and publishers will readily appreciate the importance of having their works recorded, with full length titles, on this list, which stands as a perpetual advertisement so long as the Journal lasts, and as far as it extends. The republication of the Journal in America enhances the advantages of the Bibliographical Record, on which no work can be entered, unless transmitted, free of expence, to the Editor, under sealed cover to the publishers, or in any other way most convenient to the parties concerned.

### XIV.
### TO CORRESPONDENTS.

The Medical Practice advertised in our last Number has been long since disposed of, and right happy we are to have an opportunity of making this announcement, as it has cost us more than *five pounds* in postage already. The thoughtless practice of directing letters, on such occasions, without paying the postage, is reprehensible; but we are forced to expose the conduct of

J. C. S—— Esq. of Chepstow, who lately sent a letter to the Editor, unpaid, respecting the practice abovementioned, and when the answer went back, he refused to receive the letter, which was returned to the Editor with double postage on it!

Dr. Belcher's communication is received, and will be disposed of in the way he wishes. Several communications have been received, which do not require specification, and which we have not acknowledged by letter. The writers may consider this silence, in general, as consent.

The letter of *Censor* contains some truths, but several errors and misconceptions. The mal-practices alluded to, if they exist, which we are not prepared to deny, do not come within the scope of a Medical Review. We have other and more important concerns to attend to.

Humanitas is over humane. He must be conscious that, of all Journals, the Medico-chirurgical Review is least prone to *hypercriticism.* But if *error* is to be combated by serious argument, we do not hold it a crime to treat *folly* with satire. That we should have enemies is natural—but that we have ever permitted personal feelings of irritation to mix themselves with criticisms or comments on books, we most confidently deny. We do not pretend to superior immunity from the failings of human nature—but the one in question we have ever detested, and we defy Humanitas, or any other person, to point out a single instance of it in the numerous articles of Review which have appeared in this Journal.

To several subscribers who have written to us respecting back numbers of the Journal, we have to state, that all the numbers from No. 1 to No 13 have been imported by the publishers, from the American *reprint,* which is verbatim that of the London Edition. The remaining numbers, up to 16, are daily expected from New York, where the Work is regularly republished.

Mr. Beddingfield's "Observations on Mr. Charles Bell's controversial Lecture," are received, and are under consideration. We hope to be able to review Mr. Bell's Work in our next number. It will then be the proper time to introduce the subject of Mr. Beddingfield's communication. We would advise Mr. B. however, to publish his paper in the original department of some of the Medical Journals previously, as we have no wish to interfere with the plans of other Journals, or alter *our* own.

The anonymous letter "to the Editor of the Lancet," could not have been published with propriety while the subject of it was *sub judice,* and afterwards it would be useless. Besides the whole of the writer's arguments were urged on a former occasion. The letter will be delivered to the writer on application.

### TO THE PUBLIC.

With a new year, a new volume, and a new period of publication, we have to express, (in addition to the usual compliments of the season) our thanks for the liberal encouragement accorded by the public to the New Series of this Journal. The proofs of this encouragement, are in the hands of the public for a long time past—as are also the proofs of our gratitude—not consisting entirely of kind thanks, but of *thanks in kind,* to wit, an increase of labour, and an extension of limits, far beyond the original stipulation, and far beyond the usual ratio of similar publications. As this return of remuneration has followed, not preceded, public patronage, it cannot be set down as the cause, but the effect of that encouragement. As such, we trust it will afford presumptive assurances that success will not enervate, but rather stimulate our industry, in future, as it has done in time past.

*January 1st,* 1825.

Bainbridge, Mr. Surgeon, St. Martin's Lane.
Baxter, Mr. Surg. Penton Street, Pentonville.
Beck, Dr. Edward, M.B. Ipswich, Suffolk.
Belcher, Wm. M.D. Licentiate of the Royal College of Surgeons in Ireland. Bandon.
Boyle, Mr. James, 4, Cleveland Square, St. James's.
Briscoe, John M.D. Surgeon to the Waterford Militia, Waterford.
Brown, Mr. Surgeon, Stamford Street, Blackfriars.
Burke, W. A. M. D. Mauritius.
Burrard, Mr. Henry—to South America.
Carrick, Mr. A. Surgeon, Kensington.
Cooke, Dr. Robt, Surgeon, Royal Artillery.
Cowie, Dr. South Crescent, Bedford Square.
Dill, Mr. John, Honble. East India Company's naval service.
Dray, Dr. D. Plymouth.
Filkin, Dr. Great Coram Street, Russel Square.
Fincham, Mr. Surgeon, Spring Gardens.
Forrest, Mr. Charles, Member of the Royal College of Surgeons of London, Merthyr-Tedvil, Glamorganshire.
Gillies, Dr. James, Bath.
Gregory, Mr. William, Student.
Hardman, Dr. Mark, Rue de Monsieur, à Paris
Hemmingway, Wm. Esq. Surgeon, Dewsbery, Yorkshire.
Holdsworth, Mr. Surgeon, Upper Mary-le-bone Street.
Holmes, Mr. Surgeon, Botley, Hants
Howlett, Mr. Surgeon, &c. Harrow
Johnston, Dr. John, Armagh, Ireland.
Lang, Mr. Surgeon, Highgate.
Lanphier, Simon M.D. Waterford
Library of the Royal West London Infirmary Villiers Street.
M'Cabe, Dr. Parade, Hastings.
Nimmo, Dr. John, Hutchison St. Glasgow.
Peach, Thomas, M.D. Loughborough, Leicestershire.
Pook, Mr. R. R. Surgeon, Great Ormond Street.
Porter, Mr. Charles. To East Indies.
Poynder, G. Esq. Gloucester.
Preston, W. C. Newcastle upon Tyne.
Rees, Mr. William Surgeon, Canada.
Ryan, Dr. Michael, Kilkenny.
Skelton, Dr. Princes Street, Cavendish Square.
Sutton, Rd. Member of the Royal College of Surgeons, and Licentiate of the Honble. Society of Apothecaries' Hall.
Tapp, Mr. William, Surgeon, Dorchester.
The Library of the Royal Westminster Infirmary.
Watkins, George Edward, Student, London.
Wight, Mr. A. Surg. &c. Watt's Terrace, Kent Road.
Wise, Thomas A. M.D. Surgeon, London.
Wood, Dr.

N.B. It may be observed, that Names, Books, Letters, &c. received after the 15th of December, have not appeared, or been acknowledged in this Number.

